# Sensing the heat with TRPM3

**DOI:** 10.1007/s00424-017-2100-1

**Published:** 2018-01-05

**Authors:** Joris Vriens, Thomas Voets

**Affiliations:** 1 Laboratory of Endometrium, Endometriosis and Reproductive Medicine, Department of Development and Regeneration, G-PURE, KU Leuven, Herestraat 49, box 611, 3000 Leuven, Belgium; 2 Laboratory of Ion Channel Research, Department of Cellular and Molecular Medicine, VIB Centre for Brain & Disease Research, KU Leuven, Leuven, Belgium

**Keywords:** TRP channels, TRPM3, Pain, Sensory neurons

## Abstract

Heat sensation, the ability to detect warm and noxious temperatures, is an ancient and indispensable sensory process. Noxious temperatures can have detrimental effects on the physiology and integrity of cells, and therefore, the detection of environmental hot temperatures is absolutely crucial for survival. Temperature-sensitive ion channels, which conduct ions in a highly temperature-dependent manner, have been put forward as molecular thermometers expressed at the endings of sensory neurons. In particular, several temperature-sensitive members of the transient receptor potential (TRP) superfamily of ion channels have been identified, and a multitude of in vivo studies have shown that the capsaicin-sensitive TRPV1 channel plays a key role as a noxious heat sensor. However, *Trpv1*-deficient mice display a residual heat sensitivity suggesting the existence of additional heat sensor(s). In this chapter, we provide evidence for the role of the non-selective calcium-permeable TRPM3 ion channel as an additional heat sensor that acts independently of TRPV1, and give an update of the modulation of this channel by various molecular mechanisms. Finally, we compare antagonists of TRPM3 to specific blockers of TRPV1 as potential analgesic drugs to treat pathological pain.

## Feel the heat

The ability to sense noxious heat represents an evolutionary conserved alarm system that helps to protect us from the detrimental effects of temperatures ≥ 43 °C on key biological macromolecules, and thus on the integrity of cells and tissues [3]. In healthy conditions, humans typically perceive temperatures ≥ 43 °C as painful [73]. However, under pathological conditions such as inflammation, sunburn, or tissue injury, the pain threshold is often lowered and the intensity of the heat pain response increases. This can give rise to heat hyperalgesia (an increased pain response to noxious heat), heat allodynia (when moderate temperatures evoke a pain response), and spontaneous burning pain without any obvious stimulus [3, 73]. Therefore, elucidating the cellular and molecular bases of noxious heat sensing is of great importance, not only to understand the basis of a fundamental and conserved biological process essential for survival but also to allow the development of therapies that counteract persistent pain under pathological conditions.

The detection and transmission of heat stimuli crucially depend on the activity of a multitude of ion channels in the plasma membrane of sensory nerves, including background and voltage-gated K^+^ channels that ensure a negative voltage over the plasma membrane in the absence of stimuli, voltage-gated Na^+^ channels that generate action potentials when a certain voltage threshold is crossed, and one or more depolarizing ion channels that open in response to heat such that the action potential threshold can be reached [3, 14, 73]. This latter type of ion channel is often considered as the primary molecular heat sensor. It should be noted, however, that the heat sensitivity of a sensory neuron is not solely dependent on the properties of the steeply heat-sensitive depolarizing channels, but rather on the blend of depolarizing and repolarizing ion channels, all of which exhibit at least some degree of thermosensitivity [69, 73]. As such, the actual contribution of temperature-sensitive ion channels to thermosensation is highly dependent on the cellular context, which may explain why some highly thermosensitive ion channels are also found in cell types that are not involved in thermosensory processes.

The cloning of capsaicin-sensitive cation channel TRPV1 represents a milestone in our understanding of the molecular basis of noxious heat sensing [7]. TRPV1 is a member of the *t*ransient *r*eceptor *p*otential (TRP) superfamily of cation channels, which function as tetramers built of subunits with six transmembrane domains and are thus structurally related to the large class of voltage-gated calcium, sodium, and potassium channels [21]. TRPV1 was found to be activated by temperatures exceeding ~ 42 °C and to be expressed in sensory terminals of pain-conveying sensory neurons (nociceptors), strongly suggesting that it plays a central role in the detection of noxious heat in the pain pathway [7]. Surprisingly, however, genetic ablation of TRPV1 in mice caused the expected loss of capsaicin sensitivity but only minor deficits in acute noxious heat sensing [6, 12]. In contrast, various strategies to eliminate TRPV1-expressing nociceptor neurons in mice invariably resulted in almost complete absence of heat nociception. These included studies where high doses of TRPV1 agonists such as capsaicin or resiniferatoxin were used to kill or long-term desensitize TRPV1-expressing neurons, as well as studies in which a diphtheria toxin-mediated neuronal cell death mechanism was introduced under the control of the TRPV1 promoter [39, 47]. The essential conclusions of these studies are that TRPV1-positive neurons are essential for noxious heat sensing and that these neurons must express one or more TRPV1-independent heat-sensing mechanisms.

Within the vanilloid (TRPV) subfamily of TRP channels, three other members (TRPV2–TRPV4) were found to be also steeply activated by heating, making them obvious candidates to participate in TRPV1-independent (noxious) heat sensing (for review, see [73]). However, this initial hypothesis has not been corroborated by the results from mouse knockout studies. Mice lacking TRPV2 showed unaltered responses to noxious heat, even when TRPV1 was concomitantly eliminated [45]. In the case of TRPV3 and TRPV4, initial studies suggested specific alterations in non-noxious and noxious heat sensing [34, 41]. However, follow-up studies using animals with a pure genetic background did not confirm a role for these channels in acute heat sensing [29], although a contribution to non-noxious warmth sensing in the skin cannot be fully excluded [15, 34, 36, 40, 41]. In this respect, also a more distantly related member of the melastatin subfamily of TRP channels, TRPM2, was recently put forward as sensor of non-noxious warmth [53, 61, 62]. At the cellular level, heat-induced TRPM2 activation was proposed to occur not only in sensory neurons but also in sympathetic neurons from the superior cervical ganglion and in warm-sensitive neurons of the preoptic area of the hypothalamus [53, 61]. Interestingly, stimulation of TRPM2-positive neurons in the preoptic area activates physiological processes that increase body temperature, suggesting that TRPM2 is a key thermostat involved in detecting changes in core body temperature [53]. Moreover, TRPM2-deficient mice show a deficit in behavioral assays that tests the ability to discriminate warm temperatures, but it is not fully clear at this point whether this behavioral deficit reflects a role of TRPM2 in peripheral or central thermosensation [61]. In any case, heat avoidance is unaffected in TRPM2-deficient mice, arguing against a key role for TRPM2 in noxious heat sensing [61].

## TRPV1-independent heat sensors—two hot candidates

In recent years, two ion channels have emerged as prominent candidates to act as TRPV1-independent molecular heat sensors involved in detecting acute noxious heat, namely ANO1/TMEM16A and TRPM3. Below, a brief account will be provided of the evidence linking ANO1/TMEM16A to acute heat sensing, before focusing on the main topic of this review, TRPM3.

### ANO1/TMEM16A—a calcium-activated chloride channel as heat sensor?

Anoctamin1/TMEM16A, member of a family of transmembrane proteins with 11 members (Ano1–11; TMEM16A–K), was initially identified as a chloride channel activated by a rise in intracellular calcium [5, 50, 79]. The name anoctamin refers to the *an*ionic nature of the current and the eight (Greek *oktō*) predicted transmembrane domains [28]. Since recent structural work indicates that there are actually ten transmembrane domains per Ano/TMEM16 subunit, and that most members of the family are actually not anion channels but rather phospholipid scramblases, the name anoctamin has become a misnomer [4, 78].

Intriguingly, even when intracellular calcium is strongly buffered to very low levels, Ano1/TMEM16A can be activated by heat and mediate a heat-activated current in sensory neurons, raising the possibility that it can contribute to acute heat sensing in vivo. Indeed, mice in which Ano1 was selectively eliminated in sensory neurons showed increased latencies to acute noxious heat [11, 33]. However, some caution is warranted and additional research required before we can conclude that Ano1/TMEM16A functions as a direct molecular sensor for acute heat pain. First, while there is evidence that Ano1/TMEM16A is expressed in sensory neurons, it remains to be demonstrated that the channel is present at the sensory nerve terminals in, for instance, the skin, which is an essential prerequisite for it to act as a primary heat sensor. Second, it has been shown that Ano1/TMEM16A can be functionally coupled to TRP channels including TRPV1 and TRPV4 via calcium entry, raising the possibility that in sensory neurons Ano1/TMEM16A may be primarily involved in transducing/amplifying the signal detected by TRPV1 rather than in acting as a primary stimulus detector [59, 60]. The deficit in acute heat sensing in the Ano/TMEM16A knockout animals would be equally compatible with the channel acting as a primary detector or as a stimulus amplifier. Third, the driving force for chloride currents is generally much less negative than that for sodium and calcium, and thus, the potential of Ano1/TMEM16A to cause depolarization upon channel activation is much smaller than that of calcium-permeable non-selective cation channels such as most TRP channels. In fact, the chloride equilibrium potential over the membrane of sensory nerve terminals is unknown, so it remains to be determined whether activation of a chloride conductance will cause a depolarization or rather a stabilization of the sensory nerve ending. In any case, the expression of temperature- and calcium-dependent anion channels in sensory nerves provides ample possibilities for the modulation of sensory responses under various (patho)physiological conditions [33].

### TRPM3—a heat sensor on (neuro)steroids

TRPM3 is a calcium-permeable non-selective cation channel belonging to the TRPM subfamily, with only limited sequence homology to TRPV1 [23, 44]. Whereas several splice isoforms have been identified, the TRPM3α2 isoform is by far the best characterized [43] and corresponds functionally to the TRPM3-dependent responses observed in the sensory system [74]. Therefore, in the remainder of this review, we use TRPM3 to denote the TRPM3α2 isoform.

TRPM3 is a polymodally activated channel that can be activated by both physical and chemical stimuli (for a detailed overview, see [27]). In a first study, heterologously expressed TRPM3 was shown to be activated by hypotonic cell swelling [23], but more robust channel activation was later shown using chemical ligands. The best characterized chemical activators of TRPM3 are the synthetic small molecule CIM0216, which is so far the most potent agonist of TRPM3 (EC_50_ ~ 0.77 μM) [25], and the endogenous neurosteroid pregnenolone sulfate (PS) (EC_50_ ~ 23 μM) [76]. It remains unclear whether PS can reach high-enough levels in vivo to act as a genuine endogenous TRPM3 ligand. Below, we summarize the evidence that TRPM3 acts as a heat sensor involved in noxious heat sensation in the somatosensory system.

## TRPM3 as a heat sensor in vitro and in vivo

### TRPM3 activation by heat—heterologous expression systems

HEK293T cells expressing TRPM3 exhibit robust and reversible responses to heat stimulation, both in Fura2-based calcium imaging and whole-cell patch-clamp experiments [74]. The thermal sensitivity of an ion channel can be quantified by measuring the 10° temperature coefficient (*Q*_10_), which is the fold increase in current upon a 10° temperature increment. A value of 7.2 has been determined for heterologously expressed TRPM3 [74], which is high in comparison to temperature-insensitive ion channels (*Q*_10_ < 2) [27] but relatively modest compared to some other thermosensitive TRP channels such as TRPV1, for which *Q*_10_ values > 15 have been reported [27, 71]. It should be noted here that a direct comparison of *Q*_10_ values between channels obtained in different studies is not possible, as the *Q*_10_ can vary strongly depending on the experimental settings and cellular background [70]. Notably, in lipid bilayers, it was shown that heat activation of TRPM3 is dependent on the presence of phosphatidylinositol-4,5-bisphosphate (PIP_2_) [67].

A common property of thermosensitive TRP channels is the synergistic effect of chemical agonists and thermal stimuli. For example, subactivating proton concentrations sensitize TRPV1 for heat activation [7, 65], and TRPM8-mediated menthol responses are strongly potentiated by cold [37, 46]. A similar synergism was also observed for heat and PS. The EC_50_ value for TRPM3 activation by PS is around 23 μM at room temperature (RT, 22 °C) [76]. However, PS concentrations as low as 100 nM were found to evoke robust TRPM3-mediated responses at 37 °C [74]. Such low PS concentrations are considered to be within the physiological range of plasma PS concentrations in humans, suggesting that PS may therefore act as endogenous agonist of TRPM3 in vivo [24]. Altogether, in vitro experiments using heterologously expressed TRPM3 demonstrate that it can act as a heat-activated channel that is able to integrate chemical and thermal stimuli.

### TRPM3 activation by heat—sensory neurons

In mammals, environmental thermal signals are detected by afferent neurons of the somatosensory system. The cell bodies of primary sensory neurons that innervate the skin are clustered in the trigeminal ganglia (TG) and the dorsal root ganglia (DRG). Expression of TRPM3 was found in peripheral sensory DRG and TG neurons of mice and rats, both at the mRNA and protein levels [56, 74]. Interestingly, quantitative RT-PCR studies showed that TRPM3 expression levels in sensory neurons are comparable to that of the other thermosensitive TRP channels TRPV1, TRPA1, and TRPM8 [68, 74]. Complementary studies comparing the relative mRNA expression of TRP channel genes at the single ganglion level showed consistent TRPM3 mRNA expression levels in DRGs from distinct vertebral segments [68]. At the functional level, TRPM3 expression in mice and rats was demonstrated in ~ 60% of the DRG and TG neurons in Fura2-based calcium-imaging experiments using PS as the stimulus [32, 74]. The number of PS-responding neurons was comparable to the number of capsaicin (TRPV1 agonist)- or allyl-isothiocyanate-responsive neurons (TRPA1 agonist) [74]. Further assessment illustrated that the functional TRPM3 expression is restricted to small-diameter (< 25 μm) unmyelinated somatosensory neurons, analogous to the TRPV1 and TRPA1 expression [74]. Typically, these slow-conduction, small-diameter C-fibers carry sensory information responsible for the detection of temperature and are involved in noxious heat sensing.

Indeed, a large fraction of heat-sensitive sensory neurons is also responsive to PS stimulation [74]. In sensory neurons from *Trpm3*^*−/−*^ mice, a small (around 15%) but significant reduction in the number of heat responders was observed [74]. In particular, the subgroup of heat-sensitive neurons responding to PS but not to capsaicin was ablated [74]. The relatively modest reduction of heat-responsive neurons in *Trpm3*^*−/−*^ mice may be explained by the co-expression within the same neurons of TRPM3 with TRPV1 and possibly other heat-sensitive ion channels. Indeed, the largest fraction of heat-sensitive neurons responded to both PS and capsaicin [74]. Taken together, these results suggest that the endogenously expressed TRPM3 channels in sensory neurons contribute to heat responses as one of multiple heat sensors. The high expression of TRPM3 in peripheral sensory neurons may suggest additional functions of the channel that are not primarily related to noxious heat detection. For instance, as TRPM3 was identified as a channel that can be activated by hypotonic cell swelling, a possible role in mechanosensory processes cannot be excluded (Grimm et al. 2003).

### TRPM3 activation by heat—in vivo evidence

*Trpm3*^*−/−*^ mice exhibit clear deficits in their avoidance to noxious heat, as evidenced by extended reaction latencies in the tail immersion and hot plate assays, and a reduced avoidance of the hot temperature zones in the thermal gradient and thermal preference tests [74]. Likewise, a prolonged latency in the hot plate and tail immersion test was observed in mice after systemic treatment with the TRPM3 inhibitors hesperetin, isosakuranetin, and primidone [32, 55].

The difference in heat responsiveness between wild-type and *Trpm3*^*−/−*^ mice becomes more pronounced following local injection of complete Freund’s adjuvant. Whereas this inflammatory challenge causes a significant reduction in the response latencies in wild-type mice, heat response latencies remain unaltered in *Trpm3*^*−/−*^ mice [74]. Similarly, pharmacological inhibition of TRPM3 by flavanones or primidone reduces the sensitivity of mice to noxious heat [32, 55]. Taken together, these results provide strong evidence for an in vivo involvement of TRPM3 in the detection of noxious heat.

## Molecular mechanisms of TRPM3 modulation

**Fig. 1:**
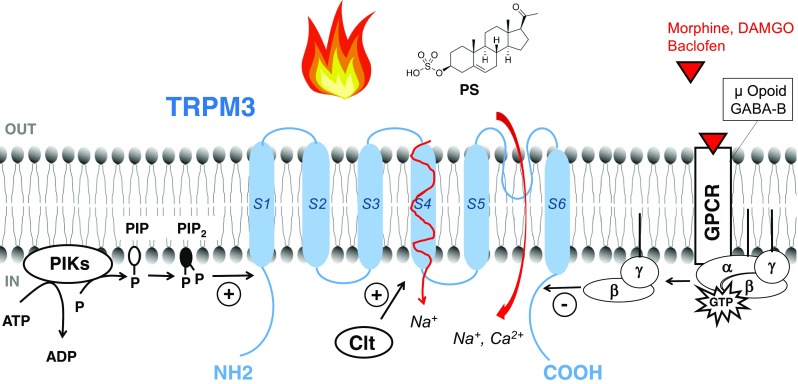
Simplified overview of TRPM3 modulation. TRPM3 can be activated by heat and the neurosteroid pregnenolone sulfate (PS). A first modulation of TRPM3 activity is regulated by phosphoinositols (PIPs). ATP restores the PIP2 level in the plasma membrane by phosphoinositol kinase activity (PIK). In addition, TRPM3 activity is regulated by G-protein-coupled receptors (GPCRs). When a GPCR like μ opioid or GABA-B receptors is activated by an agonist molecule like morphine, DAMGO, or baclofen, the heterotrimeric complex can interact with the cytosolic surface of the GPCR. After binding to GTP, the complex is dissociated into G_α_-GTP and a G_βγ_ subunit. TRPM3 activity is inhibited by direct binding to G_βγ_. A third modulator of TRPM3 is clotrimazole (Clt) that can induce the opening of a non-canonical ionic pore in the presence of PS

TRPM3 activity can be modulated via various molecular mechanisms, schematically summarized in Fig. 1.

### Phosphatidylinositol phosphates

Like many other TRP channels, TRPM3 channel activity is positively regulated by the abundant phosphoinositide phosphoinositol 4,5-biphosphate (PI(4,5)P_2_) [1, 66]. Depletion of the PI(4,5)P_2_ level in the plasma membrane decreased the activity of TRPM3 in whole-cell patch-clamp measurements and in intact cells, whereas exogenous PI(4,5)P_2_ applied to the intracellular surface of the plasma membrane returned TRPM3 activity in inside-out patches [1, 66]. Furthermore, it was demonstrated that ATP applied to the cytosolic side exhibits a strong stimulatory effect on TRPM3 activity, which requires the activity of PI-kinases resulting in the (re)synthesis of phosphatidylinositol phosphates (PIPs). Different from other TRP channels, TRPM3 discriminated little between different forms of PIP_2_ (PI(4,5)P_2_, PI(3,5)P_2_, or PI(3,4)P_2_), and its activity was more potently enhanced by (PI(3,4,5)P_3_) [1, 66].

These results provide the first potential link between TRPM3 activity and metabotropic receptors such as the histamine or bradykinin receptors, which are implicated in nociception and inflammation. Rapid depletion of PI(4,5)P_2_ by receptor-induced PLC activation may quickly suppress TRPM3 activity, whereas receptor-induced PI3-kinase activation may result in a rise in PI(3,4,5)P_3_ and thereby enhance TRPM3 activity. At this point, the consequences of TRPM3 modulation by receptor-mediated phosphoinositide metabolism for (patho)physiological heat sensing remain unclear. A study on planar lipid bilayers reported that heat-induced activation of TRPM3 occurs only in the presence of PIP_2_ [67], but how this translates to intact sensory neurons remains to be established.

### TRPM3 modulation by G-protein-coupled receptors

Recently, evidence was provided for an alternative mechanism of regulation of TRPM3 by G-protein-coupled receptors (GPCRs) [2, 13, 48]. TRPM3 channel activity induced by chemical ligands was strongly and reversibly inhibited upon stimulation of a variety of GPCRs, including the μ opioid, neuropeptide Y, and GABA-B receptors. Moreover, in HEK293 cells co-expressing the GABA_B_ or μ opioid receptors with TRPM3 channels, a complete inhibition of the Ca^2+^ responses to heat pulses was observed in the presence of the GABA_B_ receptor agonist baclofen or μ opioid receptor agonist DAMGO, respectively [2, 13]. Direct binding to the channel of the G_βγ_ subunit of the trimeric G-proteins, rather than a G_αi_- or cAMP-mediated mechanism, was identified as the molecular mechanism underlying the inhibitory effect [2, 13, 48]. Importantly, the activity of the endogenous TRPM3 in DRG neurons was also negatively modulated by G_βγ_ following activation of various GPCRs expressed in these neurons, including the μ opioid, somatostatin, NPY, and GABA_B_ receptors [2, 13, 48]. In vivo, peripheral activation of GPCRs by administration of morphine, DAMGO, PYY, or baclofen strongly attenuated TRPM3-dependent pain evoked by intraplantar PS and CIM0216 injection [2, 13, 48]. Remarkably, the activation of the same GPCRs does not strongly inhibit other TRP channels that are co-expressed with TRPM3 in DRG neurons, namely TRPV1 and TRPA1. This was supported by the fact that injection of DAMGO was ineffective in reducing the capsaicin-induced nocifensive behavior [13]. Interestingly, inhibition of μ opioid and NPY Y2 receptor activity using the respective inverse agonists naloxone and BIIE0246 augmented TRPM3 activity in DRG neurons and TRPM3-mediated pain responses in vivo, indicating that these receptors have a basal level of activity that tonically inhibits TRPM3 activity.

These data established TRPM3 channels as a privileged target of peripheral μ opioid receptors, raising the possibility that TRPM3 may be an important target in the analgesic effects of peripheral opioids. However, whereas the above-described in vitro experiments demonstrated a reduced response to heat stimulation in TRPM3-overexpressing cells, it is not yet known whether the noxious thermoreception via TRPM3 is affected by agonists of the peripheral GPCRs in vivo and whether this may contribute to the analgesic effects of opioids on inflammatory thermal hyperalgesia.

### Opening of an alternative permeation pathway

As other TRP channels, TRPM3 activation by heat or PS opens the central pore formed by transmembrane segments S5 and S6 and induces outwardly rectifying currents in TRPM3-expressing cells. The central pore of TRPM3 is highly permeable for Ca^2+^, Mg^2+^, and Zn^2+^ [43, 75] and can be blocked by the non-specific cation channel blocker La^3+^ [23, 72]. In a search for TRPM3 modulators, the antifungal compound clotrimazole (Clt) was found not only to enhance PS-activated TRPM3 currents but also to induce a distinct, inwardly rectifying current component at negative voltages. Remarkably, this inwardly rectifying current component showed biophysical properties that were clearly distinct from the canonical current through the central pore, including a strong inward rectification, low permeability to Ca^2+^, and minimal sensitivity to La^3+^ block [72]. Moreover, the inwardly rectifying current was not affected by a mutation in the central pore but abolished by mutations in the transmembrane region S4 [72]. Taken together, these findings indicate that the inwardly rectifying current is mediated via an alternative permeation pathway, distinct from the central pore. Although the nature of this alternative pathway is still obscure, it can be hypothesized that it involved the voltage-sensing domain formed by S1–S4, similar to the “omega pore” described in voltage-dependent Na^+^, K^+^, or Ca^2+^ channels, [26, 51, 52, 63, 64]. Notably, activation of the alternative permeation pathway strongly enhanced nociceptor action potential firing and exacerbated TRPM3-induced pain in vivo [72]. Note that the opening of the alternative permeation pathway is highly stimulus dependent. Whereas heat, either alone or in combination with Clt, is not able to gate the alternative permeation pathway, activation of the inwardly rectifying current by PS + Clt was strongly enhanced at 37 °C compared to RT [72]. Further research is required to establish under which (patho)physiological conditions the alternative pathway is activated in vivo.

## TRPM3 antagonists to ease the pain?

The discovery of TRPM3 as a nociceptor channel in sensory neurons, implicated in both acute pain and inflammatory heat hyperalgesia, has raised the possibility that TRPM3 activity may be pharmacologically targeted to treat pathological pain. The first preclinical studies in this respect are surprisingly encouraging. Several TRPM3 antagonists have been characterized in vitro, including secondary plant metabolites such as the flavonones naringenin (IC_50_ ~ 500 nM), liquiritigenin (IC_50_ ~ 500 nM), and isosakuranetin (IC_50_ ~ 50 nM) [55, 56], as well as approved drugs known to affect other molecular targets, including the non-steroidal anti-inflammatory diclofenac (IC_50_ ~ 6.2 μM), the antidepressant maprotiline (IC_50_ ~ 1.3 μM), and the anticonvulsant primidone (IC_50_ ~ 600 nM) [32, 57]. Interestingly, several of these compounds potently inhibit the pain response to intraplantar injection of PS, increase the latency to hot stimuli, and revert heat hyperalgesia in inflammatory or neuropathic pain models [8, 30, 32, 55]. Moreover, as described above, recent work demonstrates that peripheral opioids can inhibit TRPM3 activity in sensory neurons via a G_βγ_-mediated mechanism [2, 13, 48], raising the exciting possibility that TRPM3 inhibition may contribute to the analgesic effects of opioids. It should be noted that none of the above-mentioned pharmacological in vivo studies have included control experiments using TRPM3-deficient animals, so some caution is warranted before not strictly linking the observed effects to TRPM3 inhibition.

Whereas these results provide a strong incentive to develop better and more specific TRPM3 antagonists as potential analgesic drugs for human use, some caution is certainly warranted. In this respect, the lessons learnt from past and current projects to develop TRPV1 antagonists for the treatment of pain may form an important beacon. In the last two decades, following the cloning of TRPV1 [7] and the finding that TRPV1 knockout mice fail to develop inflammatory heat hyperalgesia [6, 12], several billions of US dollars have been invested in the (pre-)clinical development of TRPV1-targeting small molecules [42, 58]. While this endeavor has yielded a multitude of classes of TRPV1 antagonists that potently and selectively inhibit the channel in vitro and in vivo [42], to the best of our knowledge, none of these has come close to reach approval for clinical use. Three main reasons can be pointed out for the disappointing return on investment of the TRPV1 antagonist development. First, it was realized that acute pharmacological inhibition of TRPV1 can cause a significant and potentially dangerous (> 40°) increase in core body temperature [18, 20]. This was somewhat unexpected, since TRPV1 was originally described as a channel that only activates at temperatures > 42 °C [7] and TRPV1 knockout mice were found to have a normal core body temperature [6, 12]. Further research clearly indicated that the hyperthermia is an on-target effect, resulting from blockade of TRPV1 in the peripheral nervous system, and that the effect is attenuated upon repeated exposure [16, 17]. Second, patients treated with TRPV1 antagonists had a significantly compromised noxious heat response, thereby increasing the risk of accidental burns [10, 31, 35]. These two potentially dangerous undesirable side effects represented a major hurdle in the clinical development of the first generation of TRPV1 antagonists, but also fueled further research towards the development of safer, second-generation antagonists that lack a pronounced hyperthermic effect and have less effect on acute heat sensing. Third, the limited amount of human studies reporting the effects of those TRPV1 antagonists that made it to clinical studies showed either no or relatively limited clinically relevant effects [42]. These include studies where orally available TRPV1 antagonists were tested in randomized trials to treat pain after third molar extraction [49], osteoarthritis pain [38], esophageal pain in patients with gastroesophageal reflux disease [31], and pruritus [22]. Preclinical studies in animal models had firmly established that genetic ablation or pharmacological inhibition of TRPV1 prevents and reverts heat hyperalgesia [6, 12, 19, 42, 77]. However, the evidence that reducing TRPV1 activity can significantly alleviate other important components of chronic pain conditions is more variable. For instance, effects of TRPV1 antagonists on mechanical hypersensitivity are detected in some studies [19, 77] but not in others [9, 54], and cold allodynia is mostly unaffected.

If we scrutinize the (comparatively little) literature on TRPM3 antagonists with respect to these three issues (hyperthermia, risk of burn injury, and effectiveness against different pain modalities), a relatively positive picture emerges. With respect to changes in acute heat sensitivity, *Trpm3*^*−/−*^ mice or rodents treated with TRPM3 antagonists show increased withdrawal latencies [56, 74], analogous to what was found for TRPV1, so this aspect will remain an important point of attention in further drug development efforts. However, with respect to hyperthermia, several studies have shown that systemic inhibition of TRPM3 with different classes of antagonists does not affect core body temperature [30, 32, 55]. The tested antagonists inhibit TRPM3 independently of the mode of channel activation (thermal, chemical) [32, 55, 56], suggesting that TRPM3 inhibition does not affect normal thermoregulation. Finally, with respect to different pain modalities, evidence has been presented that TRPM3 antagonists alleviated mechanical, heat, and cold hyperalgesia/allodynia [8, 30], suggesting that TRPM3 antagonism may have an overall broader effect on pain sensation than TRPV1 antagonism. Nevertheless, it should be noted that further studies with a more selective TRPM3 antagonist are required before strong conclusions can be drawn regarding TRPM3 as a pain target and that it remains difficult to predict to what extent statistically significant preclinical data in animal models of pain translate towards clinically relevant pain relief in human diseases.

## Conclusions and perspectives

It is now well established that TRPM3 forms heat-sensitive ion channels in sensory neurons, where it is co-expressed with TRPV1, and that both channels are implicated in acute heat-induced pain. However, pharmacological inhibition of TRPV1 in the TRPM3-deficient mice did not fully abrogate avoidance responses to noxious heat [74], implying the existence of one or more additional molecular sensors for the detection of noxious heat in sensory neurons; the calcium-activated chloride channel Ano1/TMEM16A is currently a hot candidate.

In recent years, evidence has accumulated indicating that pharmacological inhibition of TRPM3 can alleviate acute pain as well as hyperalgesia in the context of inflammation and nerve injury. Preclinical studies in rodents are highly promising and suggest that inhibition of TRPM3 causes significant analgesia in various pain models without the undesirable side effects that hindered the clinical progress of TRPV1 antagonists. Further studies may reveal the exact mechanisms whereby TRPM3 modulates pathological pain, and establish whether the preclinical observed effects of TRPM3 antagonists translate to clinically relevant analgesia in humans, as a basis for the development of a novel class of painkillers.

## References

[1] Badheka D, Borbiro I, Rohacs T (2015). Transient receptor potential melastatin 3 is a phosphoinositide-dependent ion channel. J Gen Physiol.

[2] Badheka D, Yudin Y, Borbiro I, Hartle CM, Yazici A, Mirshahi T, and Rohacs T (2017) Inhibition of transient receptor potential melastatin 3 ion channels by G-protein betagamma subunits. Elife 6: doi: 10.7554/eLife.26147 10.7554/eLife.26147 PMC5593506 28829742

[3] Basbaum AI, Bautista DM, Scherrer G, Julius D (2009). Cellular and molecular mechanisms of pain. Cell.

[4] Brunner JD, Schenck S, Dutzler R (2016). Structural basis for phospholipid scrambling in the TMEM16 family. Curr Opin Struct Biol.

[5] Caputo A, Caci E, Ferrera L, Pedemonte N, Barsanti C, Sondo E, Pfeffer U, Ravazzolo R, Zegarra-Moran O, Galietta LJ (2008). TMEM16A, a membrane protein associated with calcium-dependent chloride channel activity. Science.

[6] Caterina MJ, Leffler A, Malmberg AB, Martin WJ, Trafton J, Petersen-Zeitz KR, Koltzenburg M, Basbaum AI, Julius D (2000). Impaired nociception and pain sensation in mice lacking the capsaicin receptor. Science.

[7] Caterina MJ, Schumacher MA, Tominaga M, Rosen TA, Levine JD, Julius D (1997). The capsaicin receptor: a heat-activated ion channel in the pain pathway. Nature.

[8] Chen L, Chen W, Qian X, Fang Y, Zhu N (2014). Liquiritigenin alleviates mechanical and cold hyperalgesia in a rat neuropathic pain model. Sci Rep.

[9] Chen Y, Yang C, Wang ZJ (2011). Proteinase-activated receptor 2 sensitizes transient receptor potential vanilloid 1, transient receptor potential vanilloid 4, and transient receptor potential ankyrin 1 in paclitaxel-induced neuropathic pain. Neuroscience.

[10] Chizh BA, O'Donnell MB, Napolitano A, Wang J, Brooke AC, Aylott MC, Bullman JN, Gray EJ, Lai RY, Williams PM, Appleby JM (2007). The effects of the TRPV1 antagonist SB-705498 on TRPV1 receptor-mediated activity and inflammatory hyperalgesia in humans. Pain.

[11] Cho H, Yang YD, Lee J, Lee B, Kim T, Jang Y, Back SK, Na HS, Harfe BD, Wang F, Raouf R, Wood JN, Oh U (2012). The calcium-activated chloride channel anoctamin 1 acts as a heat sensor in nociceptive neurons. Nat Neurosci.

[12] Davis JB, Gray J, Gunthorpe MJ, Hatcher JP, Davey PT, Overend P, Harries MH, Latcham J, Clapham C, Atkinson K, Hughes SA, Rance K, Grau E, Harper AJ, Pugh PL, Rogers DC, Bingham S, Randall A, Sheardown SA (2000). Vanilloid receptor-1 is essential for inflammatory thermal hyperalgesia. Nature.

[13] Dembla S, Behrendt M, Mohr F, Goecke C, Sondermann J, Schneider FM, Schmidt M, Stab J, Enzeroth R, Leitner MG, Nunez-Badinez P, Schwenk J, Nurnberg B, Cohen A, Philipp SE, Greffrath W, Bunemann M, Oliver D, Zakharian E, Schmidt M, and Oberwinkler J (2017) Anti-nociceptive action of peripheral mu-opioid receptors by G-beta-gamma protein-mediated inhibition of TRPM3 channels. Elife 6: doi: 10.7554/eLife.26280 10.7554/eLife.26280 PMC5593507 28826482

[14] Dubin AE, Patapoutian A (2010). Nociceptors: the sensors of the pain pathway. J Clin Invest.

[15] Fromy B, Josset-Lamaugarny A, Aimond G, Pagnon-Minot A, Marics I, Tattersall GJ, Moqrich A, Sigaudo-Roussel D (2017) Disruption of TRPV3 impairs heat-evoked vasodilation and thermoregulation: a critical role of CGRP. J Invest Dermatol. 10.1016/j.jid.2017.10.006 10.1016/j.jid.2017.10.006 29054601

[16] Garami A, Pakai E, Oliveira DL, Steiner AA, Wanner SP, Almeida MC, Lesnikov VA, Gavva NR, Romanovsky AA (2011). Thermoregulatory phenotype of the Trpv1 knockout mouse: thermoeffector dysbalance with hyperkinesis. J Neurosci.

[17] Gavva NR, Bannon AW, Hovland DN, Lehto SG, Klionsky L, Surapaneni S, Immke DC, Henley C, Arik L, Bak A, Davis J, Ernst N, Hever G, Kuang R, Shi L, Tamir R, Wang J, Wang W, Zajic G, Zhu D, Norman MH, Louis JC, Magal E, Treanor JJ (2007). Repeated administration of vanilloid receptor TRPV1 antagonists attenuates hyperthermia elicited by TRPV1 blockade. J Pharmacol Exp Ther.

[18] Gavva NR, Bannon AW, Surapaneni S, Hovland DN, Lehto SG, Gore A, Juan T, Deng H, Han B, Klionsky L, Kuang R, Le A, Tamir R, Wang J, Youngblood B, Zhu D, Norman MH, Magal E, Treanor JJ, Louis JC (2007). The vanilloid receptor TRPV1 is tonically activated in vivo and involved in body temperature regulation. J Neurosci.

[19] Gavva NR, Tamir R, Qu Y, Klionsky L, Zhang TJ, Immke D, Wang J, Zhu D, Vanderah TW, Porreca F, Doherty EM, Norman MH, Wild KD, Bannon AW, Louis JC, Treanor JJ (2005). AMG 9810 [(E)-3-(4-t-butylphenyl)-N-(2,3-dihydrobenzo[b][1,4] dioxin-6-yl)acrylamide], a novel vanilloid receptor 1 (TRPV1) antagonist with antihyperalgesic properties. J Pharmacol Exp Ther.

[20] Gavva NR, Treanor JJ, Garami A, Fang L, Surapaneni S, Akrami A, Alvarez F, Bak A, Darling M, Gore A, Jang GR, Kesslak JP, Ni L, Norman MH, Palluconi G, Rose MJ, Salfi M, Tan E, Romanovsky AA, Banfield C, Davar G (2008). Pharmacological blockade of the vanilloid receptor TRPV1 elicits marked hyperthermia in humans. Pain.

[21] Gees M, Owsianik G, Nilius B, Voets T (2012). TRP channels. Compr Physiol.

[22] Gibson RA, Robertson J, Mistry H, McCallum S, Fernando D, Wyres M, Yosipovitch G (2014). A randomised trial evaluating the effects of the TRPV1 antagonist SB705498 on pruritus induced by histamine, and cowhage challenge in healthy volunteers. PLoS One.

[23] Grimm C, Kraft R, Sauerbruch S, Schultz G, Harteneck C (2003). Molecular and functional characterization of the melastatin-related cation channel TRPM3. J Biol Chem.

[24] Harteneck C (2013). Pregnenolone sulfate: from steroid metabolite to TRP channel ligand. Molecules.

[25] Held K, Kichko T, De Clercq K, Klaassen H, Van Bree R, Vanherck JC, Marchand A, Reeh PW, Chaltin P, Voets T, Vriens J (2015). Activation of TRPM3 by a potent synthetic ligand reveals a role in peptide release. Proc Natl Acad Sci U S A.

[26] Held K, Voets T, Vriens J (2016). Signature and pathophysiology of non-canonical pores in voltage-dependent cation channels. Rev Physiol Biochem Pharmacol.

[27] Held K, Voets T, Vriens J (2015). TRPM3 in temperature sensing and beyond. Temperature (Austin).

[28] Huang F, Wong X, Jan LY (2012). International Union of Basic and Clinical Pharmacology. LXXXV: calcium-activated chloride channels. Pharmacol Rev.

[29] Huang SM, Li X, Yu Y, Wang J, Caterina MJ (2011). TRPV3 and TRPV4 ion channels are not major contributors to mouse heat sensation. Mol Pain.

[30] Jia S, Zhang Y, Yu J (2017). Antinociceptive effects of isosakuranetin in a rat model of peripheral neuropathy. Pharmacology.

[31] Krarup AL, Ny L, Astrand M, Bajor A, Hvid-Jensen F, Hansen MB, Simren M, Funch-Jensen P, Drewes AM (2011). Randomised clinical trial: the efficacy of a transient receptor potential vanilloid 1 antagonist AZD1386 in human oesophageal pain. Aliment Pharmacol Ther.

[32] Krugel U, Straub I, Beckmann H, Schaefer M (2017). Primidone inhibits TRPM3 and attenuates thermal nociception in vivo. Pain.

[33] Lee B, Cho H, Jung J, Yang YD, Yang DJ, Oh U (2014). Anoctamin 1 contributes to inflammatory and nerve-injury induced hypersensitivity. Mol Pain.

[34] Lee H, Iida T, Mizuno A, Suzuki M, Caterina MJ (2005). Altered thermal selection behavior in mice lacking transient receptor potential vanilloid 4. J Neurosci.

[35] Manitpisitkul P, Mayorga A, Shalayda K, De Meulder M, Romano G, Jun C, Moyer JA (2015). Safety, tolerability and pharmacokinetic and pharmacodynamic learnings from a double-blind, randomized, placebo-controlled, sequential group first-in-human study of the TRPV1 antagonist, JNJ-38893777, in healthy men. Clin Drug Investig.

[36] Marics I, Malapert P, Reynders A, Gaillard S, Moqrich A (2014). Acute heat-evoked temperature sensation is impaired but not abolished in mice lacking TRPV1 and TRPV3 channels. PLoS One.

[37] McKemy DD, Neuhausser WM, Julius D (2002). Identification of a cold receptor reveals a general role for TRP channels in thermosensation. Nature.

[38] Miller F, Bjornsson M, Svensson O, Karlsten R (2014). Experiences with an adaptive design for a dose-finding study in patients with osteoarthritis. Contemp Clin Trials.

[39] Mishra SK, Tisel SM, Orestes P, Bhangoo SK, Hoon MA (2011). TRPV1-lineage neurons are required for thermal sensation. EMBO J.

[40] Miyamoto T, Petrus MJ, Dubin AE, Patapoutian A (2011). TRPV3 regulates nitric oxide synthase-independent nitric oxide synthesis in the skin. Nat Commun.

[41] Moqrich A, Hwang SW, Earley TJ, Petrus MJ, Murray AN, Spencer KS, Andahazy M, Story GM, Patapoutian A (2005). Impaired thermosensation in mice lacking TRPV3, a heat and camphor sensor in the skin. Science.

[42] Moran MM, Szallasi A (2017) Targeting nociceptive TRP channels to treat chronic pain: current state of the field. Br J Pharmacol. 10.1111/bph.14044 10.1111/bph.14044 PMC5980611 28924972

[43] Oberwinkler J, Lis A, Giehl KM, Flockerzi V, Philipp SE (2005). Alternative splicing switches the divalent cation selectivity of TRPM3 channels. J Biol Chem.

[44] Oberwinkler J, Philipp SE (2014). Trpm3. Handb Exp Pharmacol.

[45] Park U, Vastani N, Guan Y, Raja SN, Koltzenburg M, Caterina MJ (2011). TRP vanilloid 2 knock-out mice are susceptible to perinatal lethality but display normal thermal and mechanical nociception. J Neurosci.

[46] Peier AM, Moqrich A, Hergarden AC, Reeve AJ, Andersson DA, Story GM, Earley TJ, Dragoni I, McIntyre P, Bevan S, Patapoutian A (2002). A TRP channel that senses cold stimuli and menthol. Cell.

[47] Pogorzala LA, Mishra SK, Hoon MA (2013). The cellular code for mammalian thermosensation. J Neurosci.

[48] Quallo T, Alkhatib O, Gentry C, Andersson DA, and Bevan S (2017) G protein betagamma subunits inhibit TRPM3 ion channels in sensory neurons. Elife 6: doi: 10.7554/eLife.26138 10.7554/eLife.26138 PMC5593501 28826490

[49] Quiding H, Jonzon B, Svensson O, Webster L, Reimfelt A, Karin A, Karlsten R, Segerdahl M (2013). TRPV1 antagonistic analgesic effect: a randomized study of AZD1386 in pain after third molar extraction. Pain.

[50] Schroeder BC, Cheng T, Jan YN, Jan LY (2008). Expression cloning of TMEM16A as a calcium-activated chloride channel subunit. Cell.

[51] Sokolov S, Scheuer T, Catterall WA (2007). Gating pore current in an inherited ion channelopathy. Nature.

[52] Sokolov S, Scheuer T, Catterall WA (2005). Ion permeation through a voltage-sensitive gating pore in brain sodium channels having voltage sensor mutations. Neuron.

[53] Song K, Wang H, Kamm GB, Pohle J, de Castro RF, Heppenstall P, Wende H, Siemens J (2016). The TRPM2 channel is a hypothalamic heat sensor that limits fever and can drive hypothermia. Science.

[54] Spicarova D, Adamek P, Kalynovska N, Mrozkova P, Palecek J (2014). TRPV1 receptor inhibition decreases CCL2-induced hyperalgesia. Neuropharmacology.

[55] Straub I, Krugel U, Mohr F, Teichert J, Rizun O, Konrad M, Oberwinkler J, Schaefer M (2013). Flavanones that selectively inhibit TRPM3 attenuate thermal nociception in vivo. Mol Pharmacol.

[56] Straub I, Mohr F, Stab J, Konrad M, Philipp SE, Oberwinkler J, Schaefer M (2013). Citrus fruit and fabacea secondary metabolites potently and selectively block TRPM3. Br J Pharmacol.

[57] Suzuki H, Sasaki E, Nakagawa A, Muraki Y, Hatano N, Muraki K (2016). Diclofenac, a nonsteroidal anti-inflammatory drug, is an antagonist of human TRPM3 isoforms. Pharmacol Res Perspect.

[58] Szallasi A, Cortright DN, Blum CA, Eid SR (2007). The vanilloid receptor TRPV1: 10 years from channel cloning to antagonist proof-of-concept. Nat Rev Drug Discov.

[59] Takayama Y, Shibasaki K, Suzuki Y, Yamanaka A, Tominaga M (2014). Modulation of water efflux through functional interaction between TRPV4 and TMEM16A/anoctamin 1. FASEB J.

[60] Takayama Y, Uta D, Furue H, Tominaga M (2015). Pain-enhancing mechanism through interaction between TRPV1 and anoctamin 1 in sensory neurons. Proc Natl Acad Sci U S A.

[61] Tan CH, McNaughton PA (2016). The TRPM2 ion channel is required for sensitivity to warmth. Nature.

[62] Togashi K, Hara Y, Tominaga T, Higashi T, Konishi Y, Mori Y, Tominaga M (2006). TRPM2 activation by cyclic ADP-ribose at body temperature is involved in insulin secretion. EMBO J.

[63] Tombola F, Pathak MM, Gorostiza P, Isacoff EY (2007). The twisted ion-permeation pathway of a resting voltage-sensing domain. Nature.

[64] Tombola F, Pathak MM, Isacoff EY (2005). Voltage-sensing arginines in a potassium channel permeate and occlude cation-selective pores. Neuron.

[65] Tominaga M, Caterina MJ, Malmberg AB, Rosen TA, Gilbert H, Skinner K, Raumann BE, Basbaum AI, Julius D (1998). The cloned capsaicin receptor integrates multiple pain-producing stimuli. Neuron.

[66] Toth BI, Konrad M, Ghosh D, Mohr F, Halaszovich CR, Leitner MG, Vriens J, Oberwinkler J, Voets T (2015). Regulation of the transient receptor potential channel TRPM3 by phosphoinositides. J Gen Physiol.

[67] Uchida K, Demirkhanyan L, Asuthkar S, Cohen A, Tominaga M, Zakharian E (2016). Stimulation-dependent gating of TRPM3 channel in planar lipid bilayers. FASEB J.

[68] Vandewauw I, Owsianik G, Voets T (2013). Systematic and quantitative mRNA expression analysis of TRP channel genes at the single trigeminal and dorsal root ganglion level in mouse. BMC Neurosci.

[69] Viana F, de la Pena E, Belmonte C (2002). Specificity of cold thermotransduction is determined by differential ionic channel expression. Nat Neurosci.

[70] Voets T (2012). Quantifying and modeling the temperature-dependent gating of TRP channels. Rev Physiol Biochem Pharmacol.

[71] Voets T, Droogmans G, Wissenbach U, Janssens A, Flockerzi V, Nilius B (2004). The principle of temperature-dependent gating in cold- and heat-sensitive TRP channels. Nature.

[72] Vriens J, Held K, Janssens A, Toth BI, Kerselaers S, Nilius B, Vennekens R, Voets T (2014). Opening of an alternative ion permeation pathway in a nociceptor TRP channel. Nat Chem Biol.

[73] Vriens J, Nilius B, Voets T (2014). Peripheral thermosensation in mammals. Nat Rev Neurosci.

[74] Vriens J, Owsianik G, Hofmann T, Philipp SE, Stab J, Chen X, Benoit M, Xue F, Janssens A, Kerselaers S, Oberwinkler J, Vennekens R, Gudermann T, Nilius B, Voets T (2011). TRPM3 is a nociceptor channel involved in the detection of noxious heat. Neuron.

[75] Wagner TF, Drews A, Loch S, Mohr F, Philipp SE, Lambert S, Oberwinkler J (2010). TRPM3 channels provide a regulated influx pathway for zinc in pancreatic beta cells. Pflugers Arch.

[76] Wagner TF, Loch S, Lambert S, Straub I, Mannebach S, Mathar I, Dufer M, Lis A, Flockerzi V, Philipp SE, Oberwinkler J (2008). Transient receptor potential M3 channels are ionotropic steroid receptors in pancreatic beta cells. Nat Cell Biol.

[77] Walker KM, Urban L, Medhurst SJ, Patel S, Panesar M, Fox AJ, McIntyre P (2003). The VR1 antagonist capsazepine reverses mechanical hyperalgesia in models of inflammatory and neuropathic pain. J Pharmacol Exp Ther.

[78] Yang H, Kim A, David T, Palmer D, Jin T, Tien J, Huang F, Cheng T, Coughlin SR, Jan YN, Jan LY (2012). TMEM16F forms a Ca2+−activated cation channel required for lipid scrambling in platelets during blood coagulation. Cell.

[79] Yang YD, Cho H, Koo JY, Tak MH, Cho Y, Shim WS, Park SP, Lee J, Lee B, Kim BM, Raouf R, Shin YK, Oh U (2008). TMEM16A confers receptor-activated calcium-dependent chloride conductance. Nature.

